# Detergent-resistant plasma membrane proteome to elucidate microdomain functions in plant cells

**DOI:** 10.3389/fpls.2013.00027

**Published:** 2013-02-22

**Authors:** Daisuke Takahashi, Yukio Kawamura, Matsuo Uemura

**Affiliations:** ^1^United Graduate School of Agricultural Sciences, Iwate UniversityMorioka, Japan; ^2^Cryobiofrontier Research Center, Faculty of Agriculture, Iwate UniversityMorioka, Japan

**Keywords:** detergent-resistant plasma membrane (DRM), microdomain, lipid raft, proteome, biotic stress, abiotic stress

## Abstract

Although proteins and lipids have been assumed to be distributed homogeneously in the plasma membrane (PM), recent studies suggest that the PM is in fact non-uniform structure that includes a number of lateral domains enriched in specific components (i.e., sterols, sphingolipids, and some kind of proteins). These domains are called as microdomains and considered to be the platform of biochemical reaction center for various physiological processes. Microdomain is able to be extracted as detergent-resistant membrane (DRM) fractions, and DRM fractions isolated from some plant species have been used for proteome and other biochemical characterizations to understand microdomain functions. Profiling of sterol-dependent proteins using a putative microdomain-disrupting agent suggests specific lipid–protein interactions in the microdomain. Furthermore, DRM proteomes dynamically respond to biotic and abiotic stresses in some plant species. Taken together, these results suggest that DRM proteomic studies provide us important information to understand physiological functions of microdomains that are critical to prosecute plant’s life cycle successfully in the aspect of development and stress responses.

## INTRODUCTION

The plasma membrane (PM) is a typical cellular membrane with selective permeability and surrounds all organelles and cellular substances. Therefore, the PM is thought to be the most important cellular membrane due to relationships to various important cellular processes including cell division, differentiation, and biotic/abiotic stress adaptation. The PM contains a variety of proteins associated with transport, signaling, cytoskeleton construction, metabolism, and stress protection in the form of transmembrane, peripheral, and lipid modified types.

Lateral distribution of these membrane proteins has been described by diffusion of each lipid and protein molecule which is proposed as fluid mosaic model (Singer and Nicolson, 1972). In addition to this hypothesis, Simons and Ikonen (1997) proposed functional microdomain of the PM. In PM microdomain hypothesis, it is considered that microdomain is organized with highly hydrophobic lipids such as sterols and sphingolipids, and specific proteins with defined functions (Brown and London, 1998, 2000; London and Brown, 2000). In animal cells, one of microdomain function is considered to be a scaffold in association with signaling complex, membrane trafficking, and transport (Simons and Ikonen, 1997; Simons and Toomre, 2000; Lingwood and Simons, 2010). Experimentally, microdomain can be obtained as non-ionic detergent-resistant membrane (DRM) fraction due to their own hydrophobic properties (Schroeder et al., 1994; Simons and Ikonen, 1997; Brown and London, 1998).

Peskan et al. (2000) reported for the first time isolation of DRM fractions from plant materials using tobacco leaves. After this report, the isolation of DRM fractions have been reported with other plant species such as tobacco, *Arabidopsis thaliana*, leek, *Medicago truncatula*, *Solanum tuberosum*, rice, oat, and rye (Mongrand et al., 2004; Borner et al., 2005; Morel et al., 2006; Laloi et al., 2007; Lefebvre et al., 2007; Krügel et al., 2008; Fujiwara et al., 2009; Minami et al., 2009; Takahashi et al., 2012). Some physiological studies showed possibilities that microdomain is involved in pollen tube tip growth, intracellular virus movement, and clathrin-independent endocytotic pathway (Liu et al., 2009; Raffaele et al., 2009; Li et al., 2012). In addition to these functions, PM microdomain may have roles in cell wall polysaccharide synthesis in hybrid aspen (Bessueille et al., 2009).

There have been attempts to identify microdomain-associated proteins for elucidation of novel microdomain-dependent regulatory mechanisms on cellular physiological processes in plant. Most of these studies were 2D or 1D electrophoresis gel-based proteomics or nano-LC-MS/MS-based shotgun proteomics using microdomain-enriched DRM fraction. In addition to DRM fraction, methyl-β-cyclodextrin (mβCD), which is known as a sterol chelator and, hence, a sterol-dependent microdomain disrupter, was used to characterize how protein was associated with the primary microdomain lipid, sterol (Kierszniowska et al., 2009). Comprehensive analyses of DRM proteomes may contribute to demonstrate the importance of lateral segregation of proteins in plant PM microdomains. Ultimately, these results may lead to new findings of plant cellular homeostasis system such as signaling machinery, transport regulation, and novel response system against perception of biotic stress such as fungal infection and abiotic stress such as drought, salt, light, nutrition, and temperature.

## DETERGENT-RESISTANT MEMBRANE FRACTION AS A BIOCHEMICAL SAMPLE FOR OBTAINING INFORMATION ASSOCIATED WITH PLASMA MEMBRANE MICRODOMAIN

**FIGURE 1:**
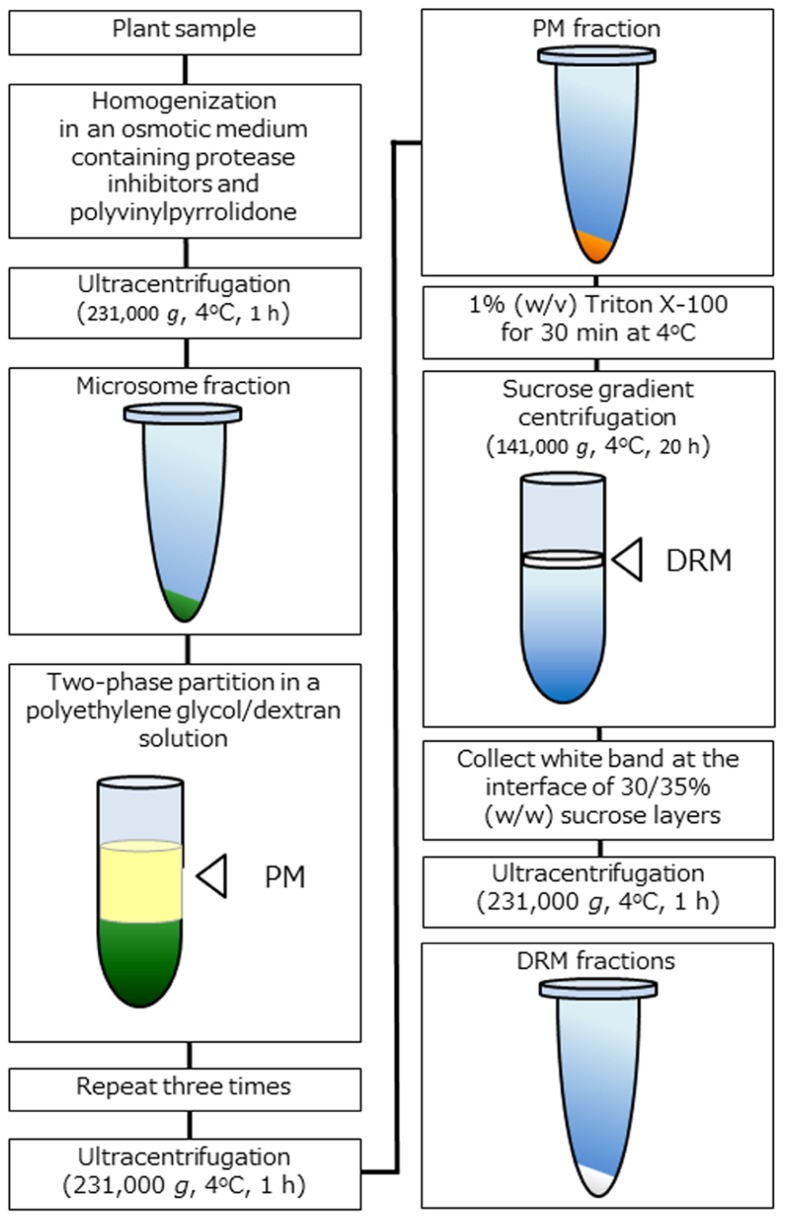
**Schematic representation of DRM extraction in plants**. Overview of the DRM extraction procedure. DRM fractions are obtained from purified PM fractions by the 1% (w/v) Triton X-100 treatment and subsequent sucrose density gradient centrifugation.

To analyze biochemical properties, the extraction of DRM fractions from the PM is considered to be the only way to prepare microdomain samples (**Figure 1**). DRM fractions were isolated from a number of plant species and tissues as described above, and the preparation protocols of DRM fractions are in general quite similar regardless of plant species. First, a highly pure PM fraction is prepared using a two-phase partition system and then treated with 1% (w/v) Triton X-100 detergent at low temperature (on ice or 4°C) for 30 min. Next, treated membrane fraction is subjected to sucrose density gradient centrifugation. After centrifugation, white band appeared at the interface of sucrose layers recovered and collected by centrifugation. Precipitated membrane fraction is suspended in a proper buffer as DRM fraction. Because unknown artificial effects might be caused due to detergent treatment at low temperature, some researchers concerned that intact microdomains that function *in vivo* are not extracted by the widely adapted preparation protocols (Tanner et al., 2011). Nevertheless, DRM fraction is a useful tool for estimating microdomain functions associated with specific components. Many microdomain-related phenomena have been elucidated in DRM and non-DRM fractions, and experiments with DRM fraction is apparently one of the most effective ways to determine specific functions in relation to microdomains in PM (Lingwood and Simons, 2007).

Proteomics approaches of DRM proteins is well-conducted in various organisms and, further, quantification of DRM and non-DRM proteins is also reported in some plant species using isotope labeling, 2D difference gel electrophoresis (2D-DIGE), and label-free quantification software (Kierszniowska et al., 2009; Minami et al., 2009; Takahashi et al., 2012). However, there are still difficulties in quantitative determination of a large number of proteins correctively using proteomic approached. This is in part because solubilization of membrane proteins including those localized in the PM as well as DRM may not be consistent in a series of experiments due to hydrophobic characteristics of the proteins and assignment of peptide fragments to the appropriate protein may not be accurate in some species for which we have not yet completed genome sequencing. It is necessary to combine another approaches (such as immunochemical and biochemical approaches) to obtain the amount of proteins in the membrane accurately.

## FUNCTION OF THE PM MICRODOMAIN

Detergent-resistant membrane proteomes have been determined in some plant species (Mongrand et al., 2004; Shahollari et al., 2004; Borner et al., 2005; Morel et al., 2006; Lefebvre et al., 2007; Fujiwara et al., 2009; Minami et al., 2009; Stanislas et al., 2009; Takahashi et al., 2012). Comparisons of DRM proteomes from these plant species indicated that DRM protein functions are very similar among plant species: DRM fractions contain many transporters, proteins associated with membrane vesicle trafficking processes and cytoskeleton such as H^+^-ATPases, aquaporins, clathrins, actins, and tubulins. Further, microscopic observations and biochemical analyses of DRM fractions or intact plant cells implied that microdomains play some functional roles in the physiological aspects. **Table 1** summarizes proteins that were found in common in some plant species on papers published so far. Localization or function of some of these proteins in distinct regions in the PM was further confirmed by additional approaches either morphologically or biochemically.

**Table 1:** Proteins identified as DRM-enriched proteins in plants by proteomic and other studies

Proteins	Deduced function	Reference	Evidence for association with microdomain
P-type ATPase	Proton transport	*Arabidopsis thaliana *(Shahollari et al., 2004; Borner et al., 2005; Minami et al., 2009) Tobacco (Mongrand et al., 2004; Morel et al., 2006; Stanislas et al., 2009) *Medicago truncatula *(Lefebvre et al., 2007) Rice (Fujiwara et al., 2009) Oat and rye (Takahashi et al., 2012)	
Aquaporin	Water channel	*Arabidopsis thaliana *(Shahollari et al., 2004; Borner et al., 2005; Minami et al., 2009) Tobacco (Mongrand et al., 2004; Morel et al., 2006; Stanislas et al., 2009) *Medicago truncatula *(Lefebvre et al., 2007) Rice (Fujiwara et al., 2009) Oat and rye (Takahashi et al., 2012)**	
Leucine-rich repeat receptor like kinase	Signaling receptor	*Arabidopsis thaliana *(Shahollari et al., 2004; Minami et al., 2009) Tobacco (Morel et al., 2006; Stanislas et al., 2009) *Medicago truncatula *(Lefebvre et al., 2007) Rice (Fujiwara et al., 2009) Oat and rye (Takahashi et al., 2012)**	
Remorin	Unknown	*Arabidopsis thaliana *(Shahollari et al., 2004; Minami et al., 2009) Tobacco (Mongrand et al., 2004; Morel et al., 2006; Stanislas et al., 2009) *Medicago truncatula *(Lefebvre et al., 2007) Oat and Rye (Takahashi et al., 2012)**	Involvement of microdomain and remorin in Potato virus X movement in tomato (Raffaele et al., 2009)
Tubulin	Cytoskeleton component	*Arabidopsis thaliana *(Minami et al., 2009) Tobacco (Mongrand et al., 2004; Morel et al., 2006; Stanislas et al., 2009) *Medicago truncatula *(Lefebvre et al., 2007) Rice (Fujiwara et al., 2009) Oat and rye (Takahashi et al., 2012)**	
NADPH oxidase (Rboh)	Response to stress	Tobacco (Mongrand et al., 2004; Morel et al., 2006; Stanislas et al., 2009) Rice (Fujiwara et al., 2009) Oat and rye (Takahashi et al., 2012)	Regulations of pollen tube tip growth through microdomain-dependent NADPH oxidase polarization in *Picea meyeri *(Liu et al., 2009)
Hypersensitive-induced reaction protein (Band 7 family proteins, flotillins)	Response to stress	*Arabidopsis thaliana *(Shahollari et al., 2004; Borner et al., 2005; Minami et al., 2009) Tobacco (Morel et al., 2006; Stanislas et al., 2009) *Medicago truncatula *(Lefebvre et al., 2007) Rice (Fujiwara et al., 2009) Oat and rye (Takahashi et al., 2012)**	Relationship with clathrin-independent endocytosis pathway in *Arabidopsis thaliana *(Li et al., 2012)
Glucan synthase	Cell wall metabolism	*Arabidopsis thaliana *(Minami et al., 2009) Tobacco (Morel et al., 2006; Stanislas et al., 2009) Rice (Fujiwara et al., 2009) Oat and rye (Takahashi et al., 2012)**	Enzymatic regulations in DRM isolated from hybrid aspen cells (Bessueille et al., 2009)

As an example of functional involvement in developmental process, Liu et al. (2009) reported the involvement of microdomain in pollen tube tip growth. Using sterol-enriched microdomains in pollen tube using one of microdomain-staining lipophilic styryl dyes, di-4-ANEPPDHQ, they clearly revealed localization of NADPH oxidase in microdomain. From the results, they suggested that one of predicted microdomain properties (i.e., clustering of specific, hydrophobic lipids and proteins) is required for NADPH oxidase activity and polarization of sterol-enriched microdomain regulates NADPH oxidase-dependent reactive oxygen species signaling. Ultimately, polar growth of pollen tube tip may be modulated by the localization of proteins in microdomain. In addition to plant pollen tip, polarization of microdomain in hyphal tip of *Candida albicans* was also observed (Martin and Konopka, 2004). These data together suggest that characteristics of microdomain are common in the function on cell polarization among various species not only plants but also microorganisms.

A recent study also suggested that microdomain is related to intracellular membrane trafficking. *Arabidopsis* Flot1 is a DRM-associated protein that was identified in DRM proteome (Borner et al., 2005). Li et al. (2012) observed that Flot1 showed patch-like localization on PM using electron microscopic technique. They further showed that Flot 1 is participated in endocytic vesicle formation but gold-conjugated antibody of Flot 1 does not co-localize with clathrin light chain. It means that Flot1 plays some roles in a microdomain-associated but clathrin-independent endocytosis pathway. Considering that RNA interference of Flot1 results in the defect of seedling development, microdomain-Flot1 mediated vesicle trafficking has important implications for seedling development such as root hair elongation regulated by vesicle trafficking (Ovecka et al., 2010).

According to protein clustering in microdomain, proteomic and subsequent enzymatic characterizations of DRM fraction from hybrid aspen cells strongly suggested the involvement of DRM in cell wall polysaccharide synthesis (Bessueille et al., 2009). DRM from hybrid aspen was enriched in glucan synthases such as callose and cellulose synthase, and, surprisingly, 73% of total glucan synthase activities of PM were detected in DRM. They concluded that microdomain is functional platform for cell wall component synthesis and controls cell morphogenesis.

Detailed analysis of *M. truncatula* DRM showed considerable differences in DRM fraction and the total PM fraction (Lefebvre et al., 2007). This study showed that free sterols, sphingolipids, and steryl glycosides are highly enriched in DRM fractions. These results are consistent with previous studies with tobacco and *A. thaliana* (Mongrand et al., 2004; Borner et al., 2005). In addition to lipids, global survey of DRM proteins were performed and revealed that signaling-, transport-, redox-, cytoskeleton-, trafficking-, and cell wall-related proteins were enriched in DRM, most of which were also found in early works of plant DRM protein identification (Mongrand et al., 2004; Shahollari et al., 2004; Borner et al., 2005; Morel et al., 2006). Proteome profiling of *M. truncatula* DRM further indicated the possible presence of microdomain-dependent redox regulation system and microdomain platform for signaling.

As described above, sterols are one of the primary components of microdomain-enriched DRM fractions in both animal and plant cells. Kierszniowska et al. (2009) applied mβCD to isolated *Arabidopsis* DRM fractions to analyze sterol-dependent enrichment of DRM proteins. mβCD is a sterol-removing cyclic oligosaccharide and mβCD treatment disrupts the organization of membrane microdomain. Proteomic analysis of the mβCD-treated and untreated DRM fractions revealed that cell wall-related and glycosylphosphatidylinositol anchored proteins (a class of lipid-modified proteins) were changed by sterol depletion. Thus, these results strongly suggest that sterol is an important factor for segregation of specific proteins into DRM fraction and PM is “phase-separated” to form specific domains (i.e., sterol-enriched microdomains, Xu et al., 2001). As shown in these studies, proteome analysis has been used for estimating microdomain functions in plant cells for the past decades and, therefore, greatly contributed to elucidation of microdomain-associated physiological functions in plant cells.

## DRM PROTEOME ON BIOTIC STRESS RESPONSE

Plant proteomic studies for elucidating microdomain function have been carried out intensively in the research area of plant–pathogen interactions. The possibility of lipid microdomain-pathogen interactions was first reported by Bhat et al. (2005). The authors suggest that fungal pathogen (*Blumeria graminis* f.sp. *hordei*) recognizes barley *mildew resistance locus o* (*Mlo*) that seems to re-localize as microdomain-like structure at pathogen invasion site. In addition to pathogen infection, plant immune responses against biotic stress may be supported by functional microdomain. Fujiwara et al. (2009) successfully identified 192 proteins from DRM proteome analysis in rice suspension cultured cells that were pre-transformed with constitutively active OsRac. OsRac1 is one of the Rac/Rop GTPase family proteins and regulates rice immunity as a key regulator (Kawasaki et al., 1999; Ono et al., 2001; Wong et al., 2004; Lieberherr et al., 2005; Kim et al., 2012). Shift of OsRac1 to DRM fractions was found after elicitor treatment. At the same time, DRM proteome suggests that microdomain exists as platform for rice innate immunity. Actually, *receptor-like kinases *(*RLK*), disease resistance proteins and band7 family proteins, members of disease-related proteins, were detected in rice DRM fractions as well as some other plant species (Borner et al., 2005; Morel et al., 2006; Fujiwara et al., 2009; Minami et al., 2009). Interactions between OsRac1 and those proteins may occur during initial immunity process against biotic stimuli.

Mongrand et al. (2004) also suggest from proteomics of DRM that microdomain isolated as DRM fractions has important functions in plant defense responses because tobacco DRM proteome contains a variety of defense-related proteins such as remorin, NtrbohD, and Ntrac5. Some physiological studies further indicated that DRM-enriched proteins are associated with plant–pathogen interactions. Remorin is the most characterized and a representative DRM protein and Raffaele et al. (2009) reported interesting results that remorin is associated with intercellular virus movement. Solanaceae remorin was fractionated into DRM fraction, which is also reported in tobacco DRM proteomics (Mongrand et al., 2004; Morel et al., 2006; Stanislas et al., 2009) as well as oat and rye proteomics (Takahashi et al., 2012). Interestingly, the distribution of remorin on the PM was represented as patch-like patterns and disappeared when mβCD was added to the sample. These results strongly suggest that DRM fractions partly reflect intact microdomain. Raffaele et al. (2009) also showed that remorin is localized in plasmodesmata and its accumulation levels affect cell-to-cell transfer of *Potato virus X *(PVX) through plasmodesmata. Detailed analysis of DRM proteome against elicitor signaling in tobacco BY-2 cells revealed that the DRM enrichment of cell trafficking related proteins (dynamins) and a signaling protein (14-3-3 protein) altered after cryptogein treatment (Stanislas et al., 2009). These studies clearly indicate that DRM proteomics has potential to find new factors of elicitor signaling pathway and their functions in plants, and DRM proteome has methodological significance in approach for findings of novel microdomain functions on plant pathology.

## DRM PROTEOME ON ABIOTIC STRESS RESPONSE

Abiotic stress response and adaptation mechanism in association with microdomain is not well characterized. The only study showing changes of DRM compositions in response to abiotic stimuli was with *Arabidopsis* leaves reported by Minami et al. (2009). They performed *Arabidopsis* DRM proteomic analysis to find the possibilities of microdomain functions for adaptation to freezing temperature. Plants can increase survival at severe freezing temperatures by sensing non-freezing low temperature and subsequently reconstituting cellular processes (called as cold acclimation; Guy, 1990; Sharma et al., 2005). Although there are a number of papers revealing considerable changes of PM compositions during cold acclimation (Uemura and Yoshida, 1984; Lynch and Steponkus, 1987; Webb et al., 1994; Kawamura and Uemura, 2003; Uemura et al., 2006), analysis of DRM compositions during cold acclimation was conducted in very few studies. Using a combination of 1D sodium dodecyl sulfate polyacrylamide gel electrophoresis (SDS-PAGE), 2D-DIGE and Liquid chromatography-tandem mass spectrometry (LC-MS/MS) and Matrix-assisted laser desorption/ionisation-time of flight mass spectrometry (MALDI-TOF/MS) analysis, Minami et al. (2009) demonstrated that proteomic profiles of DRM fractions altered significantly during cold acclimation. The cold acclimation-responsive proteins include synaptotagmin protein homolog, tubulin and P-type ATPase. Each protein is considered to have important roles in cold acclimation process from previous studies. For example, synaptotagmin homolog SYT1 was identified in DRM and increased after cold acclimation. SYT1 is related to calcium-dependent PM resealing (or repairing) process when PM destruction occurs due to freeze-induced mechanical stress imposed by extracellular ice formation (Yamazaki et al., 2008). In addition to SYT1, other membrane fusion-related proteins such as syntaxin were identified in oat and rye DRM (Takahashi et al., 2012). Disassembly of microtubule consisting of tubulins is suggested to be important for inducing cold acclimation process (Abdrakhamanova et al., 2003). Enhancement of ATPase activity in PM during cold acclimation is one of the well-known reactions in some plant species (Ishikawa and Yoshida, 1985; Martz et al., 2006).

How interactions between these proteins and microdomain properties affect cold acclimation processes, however, is still to be elucidated. We need to conduct additional physiological and microscopic experiments to understand responsiveness of microdomain and/or DRM proteins to cold acclimation. We have evidence from proteomic studies that there are several interesting abiotic stress-related proteins in DRM fractions such as RLKs, aquaporins, heat shock proteins, actins, and clathrins in various plants (Mongrand et al., 2004; Borner et al., 2005; Morel et al., 2006; Takahashi et al., 2012). To elucidate their contribution to abiotic stress sensing, signaling, and response, comprehensive proteomic analyses such as protein–protein interactions and post-translational modifications of the proteins would be necessary and expected.

## FUTURE PERSPECTIVE

Proteomic analyses of DRM fractions have been conducted and provided information for suggestive but important functions of PM microdomain in plants. Several physiological studies using both intact cells and isolated membrane fractions supported implications derived from proteomic analyses with DRM and added further interesting information on the roles of membrane microdomains. However, evidence of functional roles of microdomains in the PM is in a large part lacking. Now we are entering in next phase for elucidating microdomain characteristics and functions in plants. We need to consider morphology and dynamics of microdomains, physical and chemical state of PM proteins in microdomains from the perspective of post-translational modifications and molecular ultrastructure, and ultimately functional significance of microdomains in various events in plant’s life. Development of microscopic and biochemical techniques, such as single-molecule tracking and artificial membrane system, will help us to understand physiological roles of microdomain in plant cells.

Plants are immobile and, thus, perception and response to environmental stimuli are quite important for plant’s life. The PM is thought to be the primary cellular compartment of these reactions because it surrounds intracellular organelles and the cytoplasm and transduces extracellular stimuli to the specific components in the cell. Microdomain is expected to play important roles in these processes. Thus, proteomic approaches will further provide useful information for understanding plant physiological responses and microdomain significance in the future.

## Conflict of Interest Statement

The authors declare that the research was conducted in the absence of any commercial or financial relationships that could be construed as a potential conflict of interest.
